# Supplementary material for: Prokaryotic nanocompartments form synthetic organelles in a eukaryote
Source: Nat Commun. 2018 Apr 3;9:1311. doi: 10.1038/s41467-018-03768-x (PMC5882880; doi:10.1038/s41467-018-03768-x)
Supplement: Supplementary file 1 — Supplementary Information [file 41467_2018_3768_MOESM1_ESM.pdf]

Prokaryotic nanocompartments form synthetic organelles in a eukaryote

Lau et al.

Supplementary Methods

Information regarding cloned plasmids:

Strains containing cargo proteins on a 2µ plasmid were used only for SDS-PAGE analysis, to maximize loading of the cargo into encapsulins and hence visibility on a gel.

- pENC1 (EncA on 2µ plasmid)

The encapsulin gene from *Myxococcus xanthus* was cloned into a 2µ plasmid backbone pAG423GAL-ccdB (Addgene #14149) digested with SpeI/XhoI, placing the gene between the GAL1 promoter and the CYC1 terminator.

We note that the EncA protein used in this study was derived from the sequence found in the NCBI database entry MXAN_3556 for the encapsulin gene. McHugh *et al*.[1] found that the native EncA protein is actually seven amino acids shorter than the NCBI annotation (GenBank CP000113.1). Nevertheless, we were still able to produce functional encapsulin with the additional seven amino acids present.

- pENC2 (TDH3p + mNeon-TP + synthetic terminator on 2µ plasmid)

The TDH3 promoter and mNeonGreen-GGSGGS-TP construct was cloned into the backbone of plasmid pENC1 digested with EagI/PsiI. The synthetic terminator sequence was obtained from previous work by Guo and Sherman[2]. The targeting peptide (TP) sequence was obtained from the C-terminus of the EncC protein (MXAN_4464) from *M. xanthus*.

- pENC3 (TDH3p + mNeon-PEST-TP + synthetic terminator on 2µ plasmid)

The destabilized mNeon construct was cloned in an analogous manner to pENC2. The PEST sequence was derived from the C-terminal PEST motif of the CLN2 protein in yeast.

- pENC4 (TDH3p + Ven1-TP + synthetic terminator and TEF1p + Ven2-TP + ENO2t on 2µ plasmid)

The split-Venus constructs were cloned in an analogous manner to pENC3. The design of the split-Venus system was obtained from previous work by Kodama and Hu[3], using the VN155 (I152L) and VC155 constructs.

Information regarding linear constructs for genomic integration:

Strains where the cargo proteins were integrated as a single copy into the yeast genome were used for all fluorescence and enzymatic assays.

The ARO10 constructs were codon re-optimized to avoid any chance of homologous recombination with the endogenous copy of ARO10.

Sequences for the following linear constructs are included in the accompanying SI file:

- mNPEST-TP (TDH3p-mNeon-PEST-TP-SYNt-KanMX4)
- mNPEST (TDH3p-mNeon-PEST-SYNt-KanMX4)
- Ven-TP (TDH3p-VenN-TP-ENO2t-TEF1p-VenC-TP-SYNt-KanMX4)
- Ven-NoTPN (TDH3p-VenN-ENO2t-TEF1p-VenC-TP-SYNt-KanMX4)

- Ven-NoTPC (TDH3p-VenN-TP-ENO2t-TEF1p-VenC-SYNt-KanMX4)
- ARO10-TP (TEF1p-ARO10-TP-SYNt-KanMX4)
- ARO10-NoTP (TEF1p-ARO10-SYNt-KanMX4)

Media formulations:

- Synthetic defined His-dropout media (SD-His) – 0.2% His-dropout mix, 0.67% yeast nitrogen base with ammonium sulfate, 2% D-glucose
- Induction media – identical to synthetic defined His-dropout media, except replacing glucose with 2% raffinose and 1% galactose

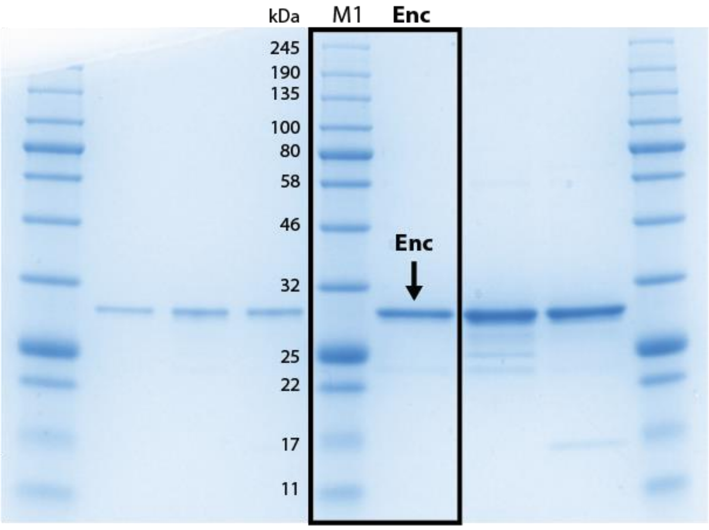

Supplementary Figure 1. Full uncropped gel corresponding to Figure 1b in the main text. Please note that the full gel for Figures 1c, 2f, and 3f is shown in Supplementary Figure 2. M1 = Color Prestained Protein Ladder, Broad Range (11-245 kDa, NEB). Enc = encapsulin protein.

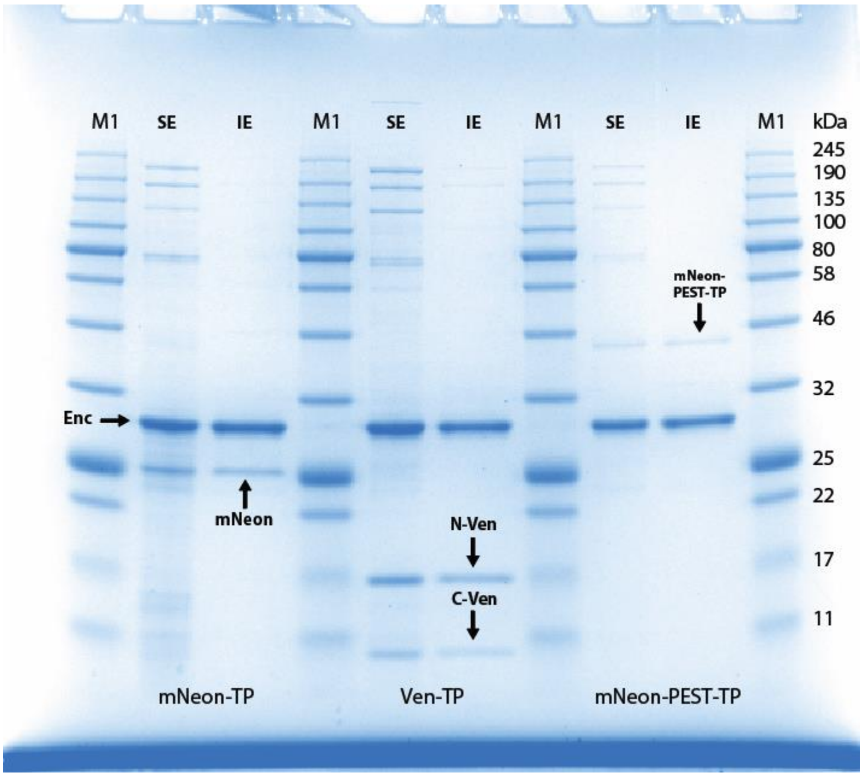

Supplementary Figure 2. FPLC purification of encapsulins as analyzed by SDS-PAGE. The various cargo types for each encapsulin (Enc) are labelled with arrows (mNeon for the mNeon-TP cargo, N-Ven and C-Ven for the split Venus Ven-TP components, and mNeon-PEST-TP for the destabilized mNeonGreen). There are minor impurity bands that are not completely removed after size-exclusion chromatography, but these are subsequently removed by ion-exchange chromatography. M1 = Color Prestained Protein Ladder, Broad Range (11-245 kDa, NEB). SE = size-exclusion, IE = ion-exchange.

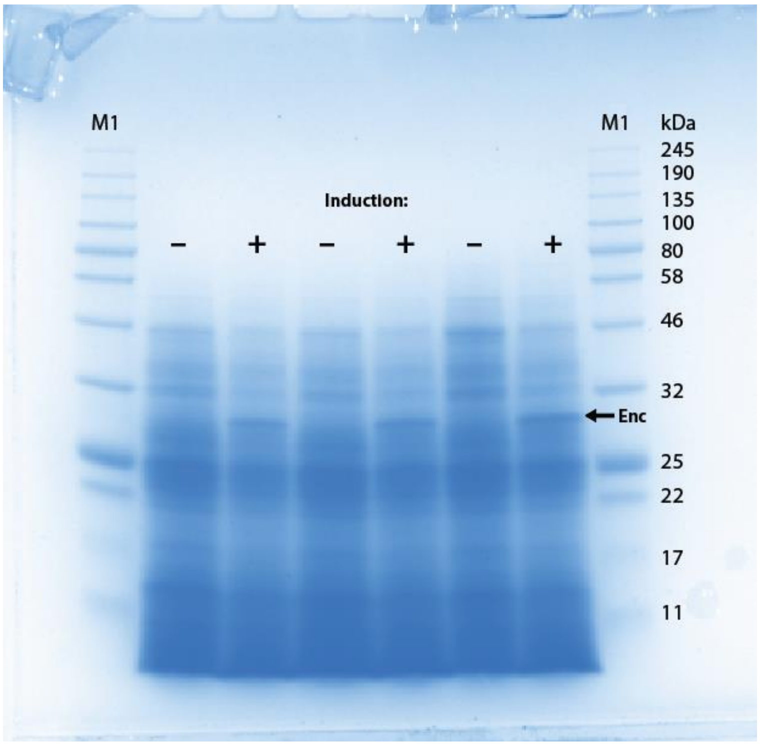

Supplementary Figure 3. Induction of *M. xanthus* encapsulin protein expression. An induction band (position indicated by arrow) is clearly visible by SDS-PAGE for cells grown in galactose induction media. The gel shows the protein content of whole cell lysates of three biological replicates grown either in galactose induction media (plus sign), or glucose non-induction media (minus sign). M1 = Color Prestained Protein Ladder, Broad Range (11-245 kDa, NEB).

Protein and Peptide Details for reference YH1andEnc **Analyzing /database/gfyuser/rtomaino/64887.fasta**

>YH1andEnc

MPLEPHFMPD FLGHAENPLR EEEWARLNET VIQVARRSLV GRRILDIYGP LGAGVQTVPY DEFQGVSPGA VDIVGEQETA MVFTDARKFK TIPIIYKDFL
LHWRDIEAAR THNMPLDVSA AAGAAALCAQ QEDELIFYGD ARLGYEGLMT ANGRLTVPLG DWTSPGGGFQ AIVEATRKLN EQGHFGPYAV VLSPRLYSQL
HRIYEKTGVL EIETIRQLAS DGVYQSNRLR GESGVVVSTG RENMDLAVSM DMVAAYLGAS RMNHPFRVLE ALLLRIKHPD AICTLEGAGA TERR

Legend

THISREFERE NCE UNMATCHED OTHERREFER ENCES

32487.56 Da	Protein Coverage		Highlight Residues	
294 AA	174 AA	**59.18 %**	K	C
			R	D
			E	

Matches	All References	This Reference
Total:	307	307
Mod-unique:	23	23
Sequence-unique:	21	21

Protein and Peptide Details for reference YH1andEnc **Analyzing /database/gfyuser/rtomaino/64887.fasta**

>YH1andEnc

MPLEPHFMPD FLGHAENPLR EEEWARLNET VIQVARRSLV GRRILDIYGP LGAGVQTVPY DEFQGVSPGA VDIVGEQETA MVFTDARKFK TIPIIYKDFL
LHWRDIEAAR THNMPLDVSA AAGAAALCAQ QEDELIFYGD ARLGYEGLMT ANGRLTVPLG DWTSPGGGFQ AIVEATRKLN EQGHFGPYAV VLSPRLYSQL
HRIYEKTGVL EIETIRQLAS DGVYQSNRLR GESGVVVSTG RENMDLAVSM DMVAAYLGAS RMNHPFRVLE ALLLRIKHPD AICTLEGAGA TERR

Legend

THISREFERE NCE UNMATCHED OTHERREFER ENCES

32487.56 Da	Protein Coverage		Highlight Residues	
294 AA	216 AA	**73.47 %**	K	C
			R	D
			E	

Matches	All References	This Reference
Total:	248	248
Mod-unique:	23	23
Sequence-unique:	19	19

Supplementary Figure 4. Mass spectrometry on the encapsulin protein. Protein sequences determined by in-gel digestion and LC/MS/MS analysis of bands extracted from SDS-PAGE gels was conducted by the Taplin Mass Spectrometry Facility at Harvard Medical School. The figure shows a summary of LC/MS/MS analysis, with coverage of both N- and C-termini of the protein.

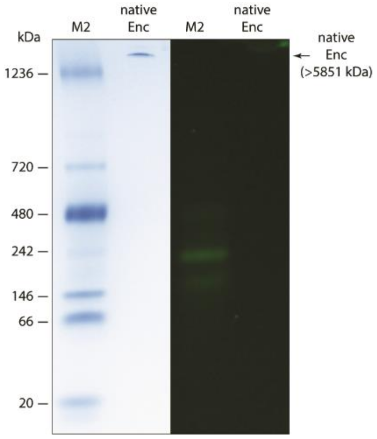

Supplementary Figure 5. Native PAGE of empty encapsulins. Native PAGE shows a high molecular weight band under Coomassie staining (left), but no in-gel fluorescence (right). This is the control to demonstrate that the encapsulins themselves are not auto-fluorescent. Enc = encapsulin. M2 = NativeMark Unstained Protein Standard (ThermoFisher).

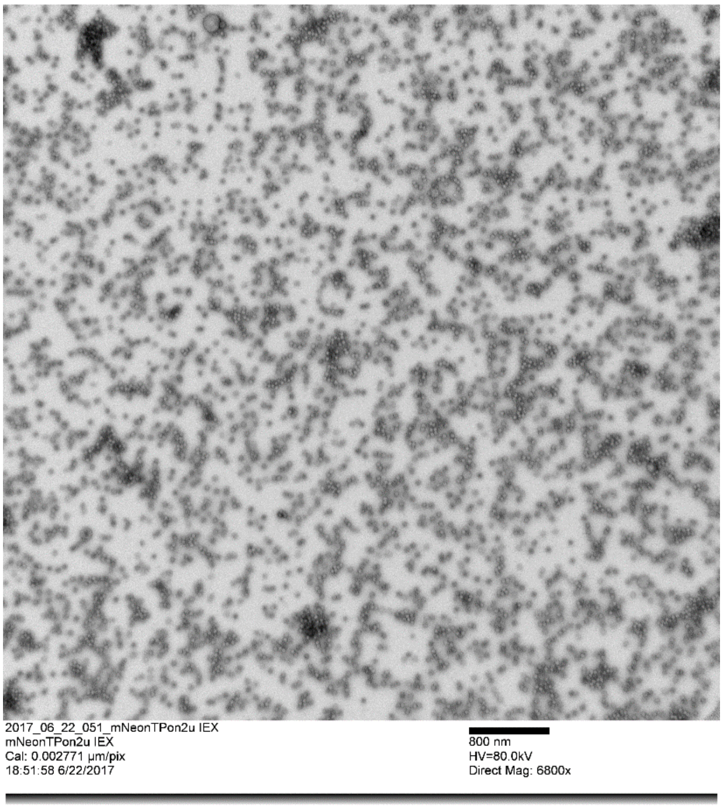

Supplementary Figure 6. An example of a low-magnification TEM image of encapsulin loaded with mNeon-TP, displaying monodispersity and uniformity across the sample. The particles were isolated from yeast and purified by both size-exclusion and ion-exchange chromatography prior to imaging. Scale bar represents 800 nm.

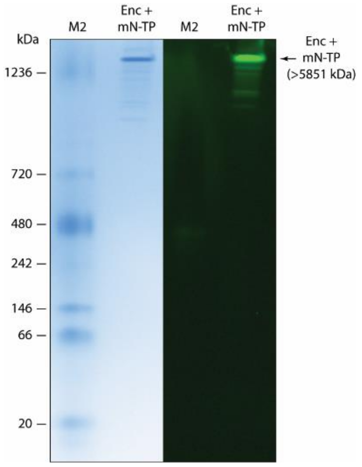

Supplementary Figure 7. Native PAGE of encapsulin with mNeon-TP cargo. Native PAGE shows a high molecular weight protein assembly band by Coomassie staining (left), accompanied by in-gel fluorescence from the associated cargo (right). Enc = encapsulin. mN-TP = mNeonGreen with targeting peptide. M2 = NativeMark Unstained Protein Standard (ThermoFisher).

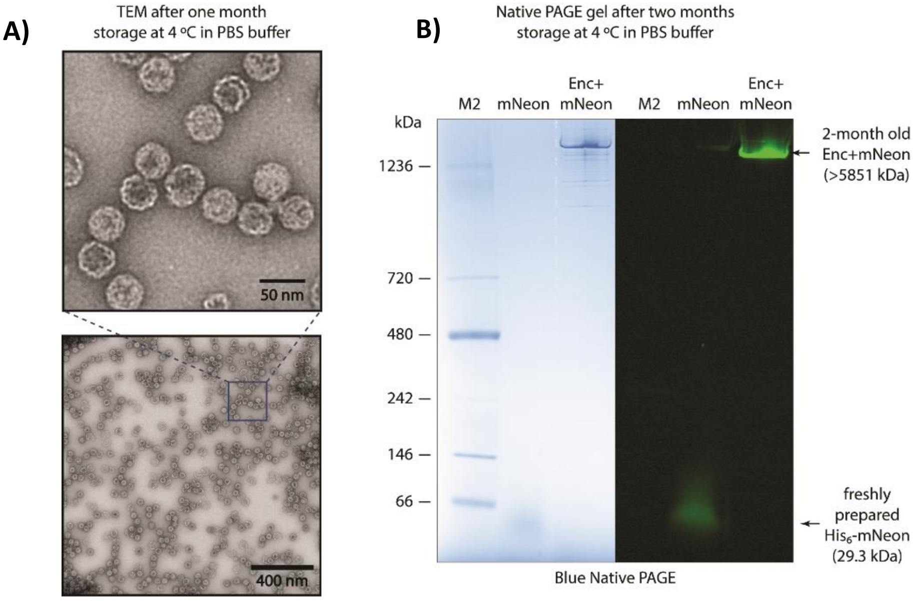

Supplementary Figure 8. Long term storage of encapsulin samples. Blue native PAGE and TEM images of encapsulins loaded with mNeon-TP after long term storage. After purification, encapsulins were concentrated to approximately 0.5 mg/mL in the last FPLC elution buffer (Tris pH 8, approximately 300 mM NaCl). Samples were stored at 4 °C for two months prior to analysis. A) The encapsulins retain the same appearance in TEM, indicating minimal degradation. B) Distinct bands for the encapsulin can also be seen, with the mNeonGreen cargo also retained inside the encapsulin assembly (Enc+mNeon band), in comparison to the free mNeonGreen (His_6-mNeon band). M2 = NativeMark Unstained Protein Standard (ThermoFisher).

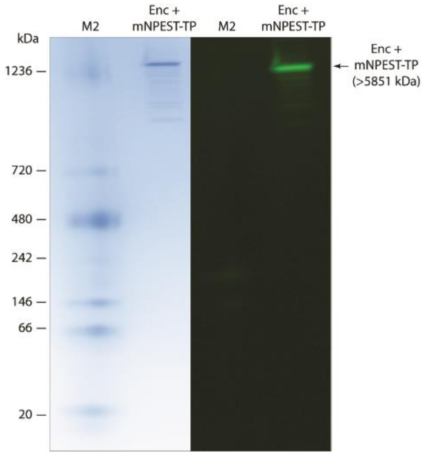

Supplementary Figure 9. Native PAGE of encapsulin with the destabilized mNeon-PEST-TP cargo. Native PAGE shows a high molecular weight protein assembly band by Coomassie staining (left), accompanied by in-gel fluorescence from the associated cargo (right). Enc = encapsulin. mNPEST-TP = mNeonGreen with PEST sequence and targeting peptide. M2 = NativeMark Unstained Protein Standard (ThermoFisher).

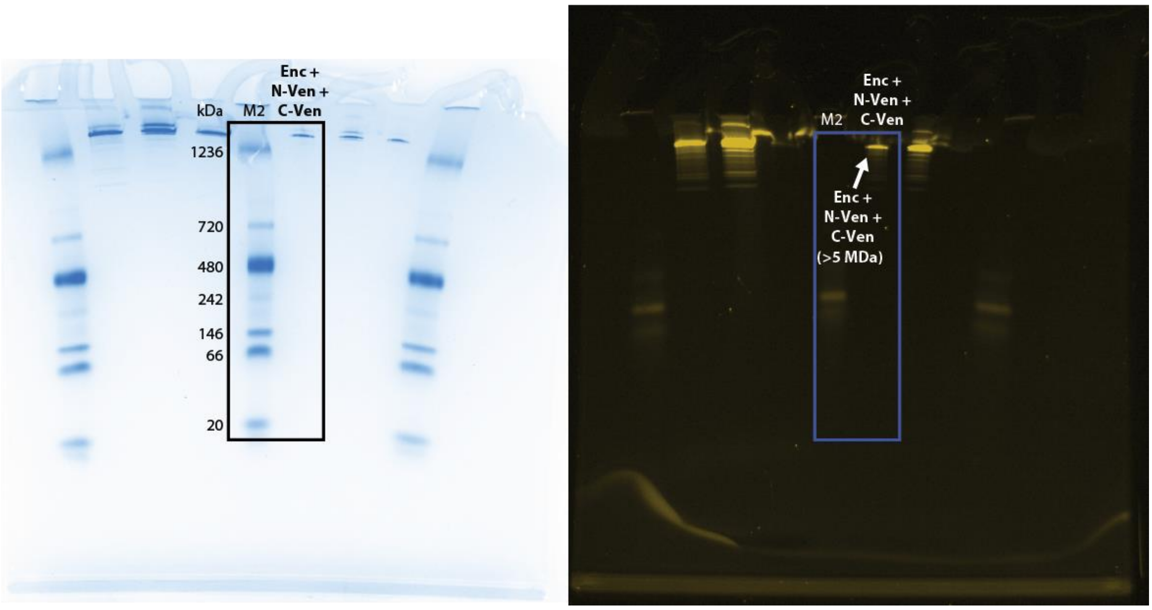

Supplementary Figure 10. Full uncropped gels corresponding to Figure 3e in the main text. M2 = NativeMark Unstained Protein Standard (ThermoFisher). Enc = encapsulin protein. N-Ven = N-terminal portion of split Venus. C-Ven = C-terminal portion of split venus.

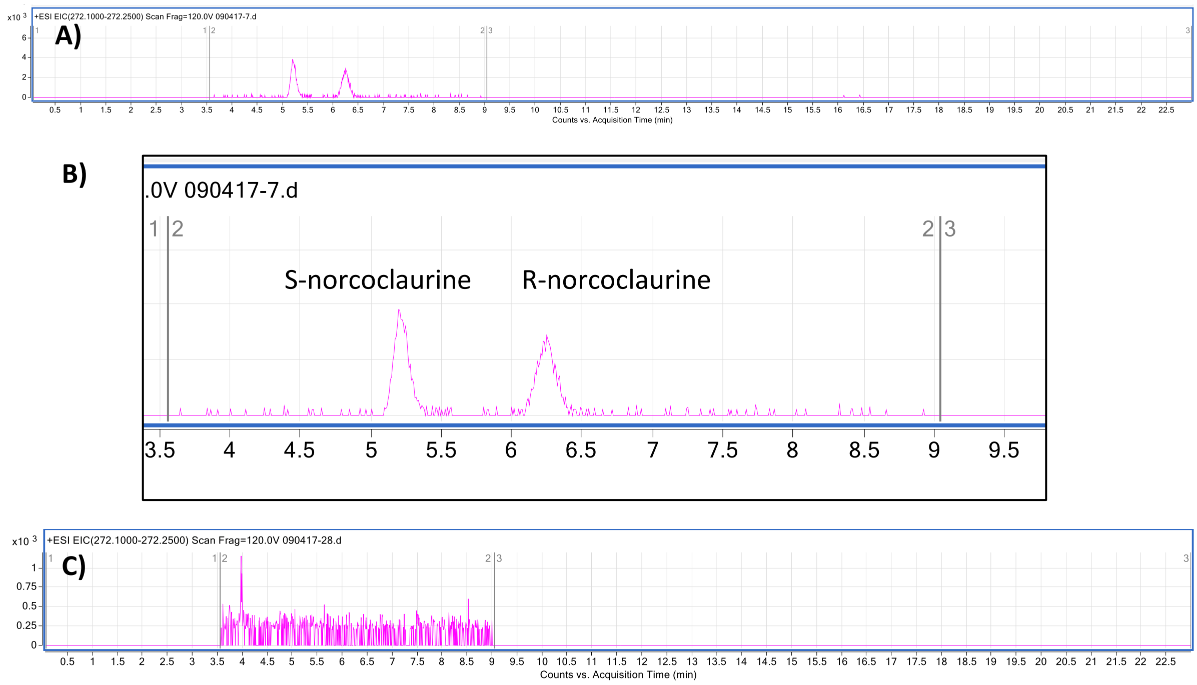

Supplementary Figure 11. Extracted ion chromatograms of the enzymatic reaction product. A) Purified encapsulins co-expressed with Aro10p-TP, B) a zoom-in of the chromatogram, and C) Aro10p with no TP. The two peaks for norcoclaurine refer to the two enantiomers that are produced in a racemic mixture by the Pictet-Spengler reaction of 4-HPAA with dopamine. Strains used for the catalytic nanoreactor experiments were Enc+ARO10-TP and Enc+ARO10. Encapsulins were isolated from these strains as described in Supplementary Table 1, doing only one chromatography purification step (gel-filtration without ion-exchange).

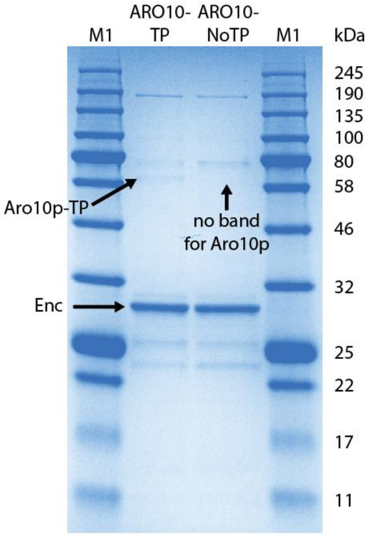

Supplementary Figure 12. SDS-PAGE comparison of encapsulins co-expressed with Aro10p-TP and Aro10p (no TP). Aro10p (73.2 kDa) only co-purifies with the encapsulin when the TP is present. We note that these samples were only purified by gel-filtration (and not subsequent ion-exchange), so minor impurity bands remain in both samples, although these do not have any enzymatic activity. Also, as ARO10-TP is only present as a single copy, the band intensity is much lower than for examples where the cargo is expressed from a plasmid. M1 = Color Prestained Protein Ladder, Broad Range (11-245 kDa, NEB).

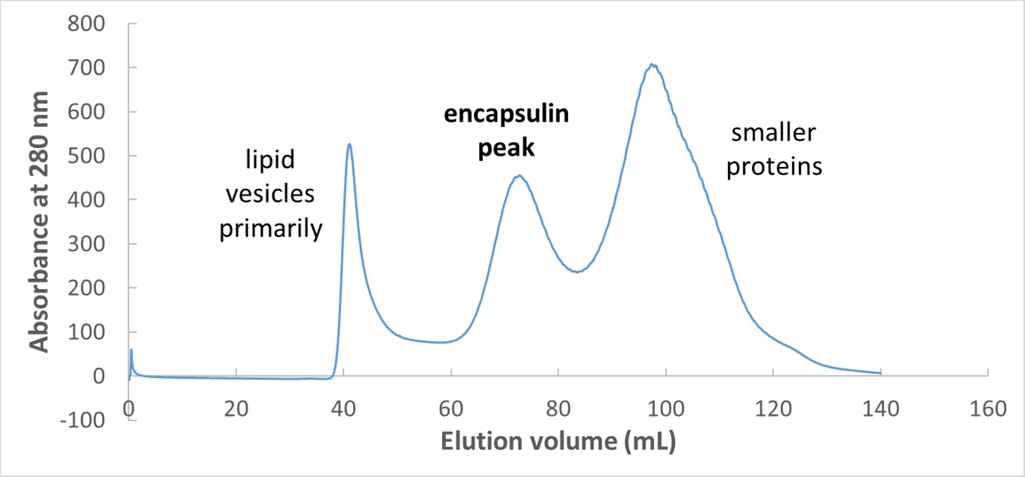

Supplementary Figure 13. Example FPLC chromatograph of size-exclusion chromatography purification of encapsulins. The encapsulin peak elutes at ~65-85 mL. Size-exclusion chromatography is the first column-based step in the purification process, leading to reasonably pure but not completely homogeneous sample.

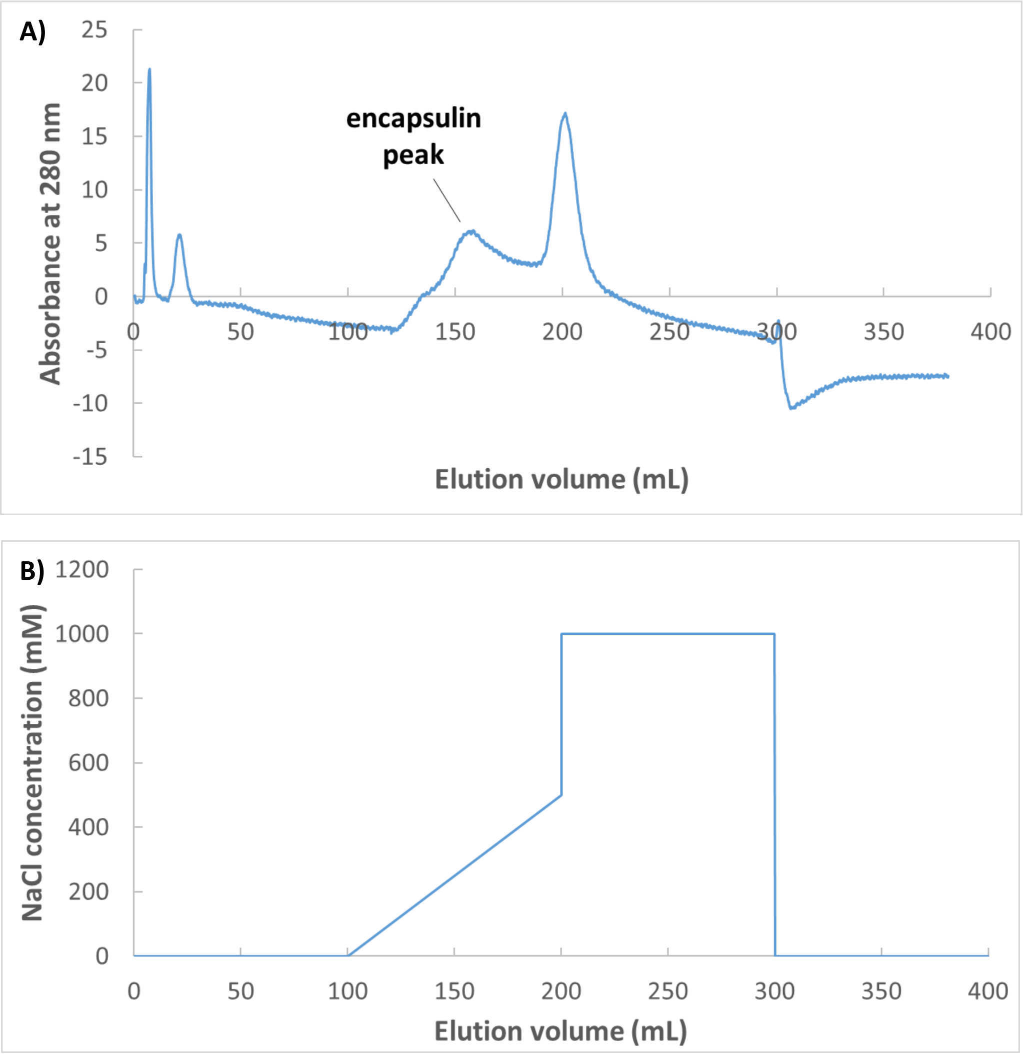

Supplementary Figure 14. A) Example FPLC chromatograph of ion-exchange chromatography purification of encapsulins, with the encapsulin peak eluting at 145-170 mL. This is the second column-based step in the purification process, leading to completely homogeneous encapsulin samples. B) The salt gradient used for the ion-exchange process, with Tris pH 8 as the base buffer.

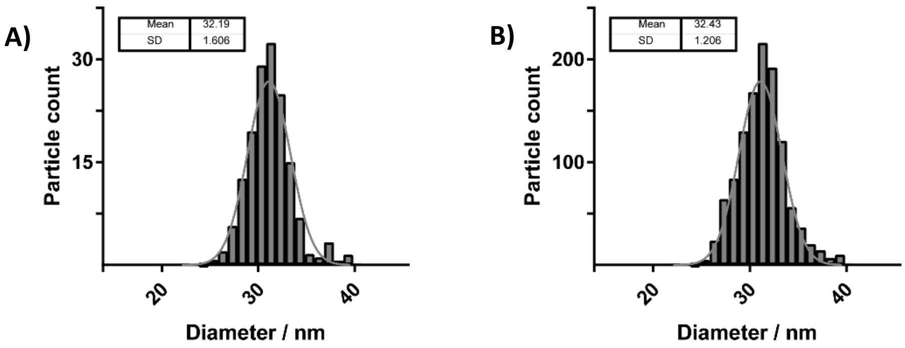

Supplementary Figure 15. Particle-size histograms for encapsulins purified from yeast. Histograms were obtained by analysis of the TEM images in Figure 1B (Panel A) and Supplementary Figure 6 (Panel B), showing that the encapsulins have the expected diameter of 32 nm. To determine the average diameters of encapsulin particles, TEM micrographs were analyzed using the open source image processing package Fiji based on ImageJ 1.51f. Micrographs were converted to 8-bit binary images, thresholded and processed using the particle analyzer plugin. The diameters reported are based on Fiji Feret diameter output values.

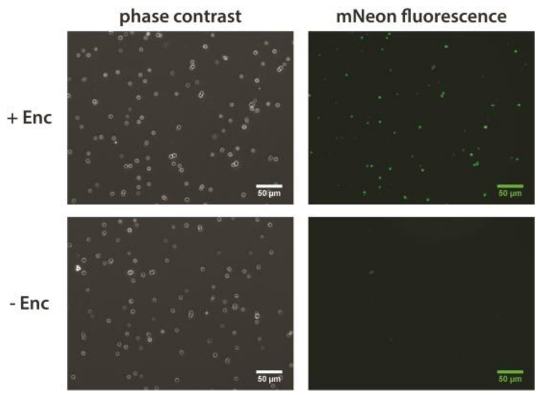

Supplementary Figure 16. Lower magnification fluorescence microscopy images of live yeast cells containing mNPEST-TP. These images accompany the more magnified images found in Figure 2C in the main text. Higher levels of fluorescence, and hence cargo, are observed when encapsulin is present, indicating that encapsulin is stabilizing its cargo in its interior. Scale bar represents 50 µm.

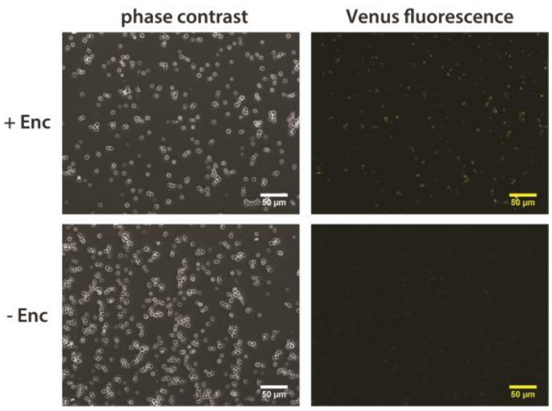

Supplementary Figure 17. Lower magnification fluorescence microscopy images of live yeast cells containing the split Venus system. These accompany the more magnified images found in Figure 3D in the main text. Higher levels of fluorescence only in the presence of encapsulin indicate that the encapsulin is co-localizing the split Venus cargo components. Scale bar represents 50 µm.

A)

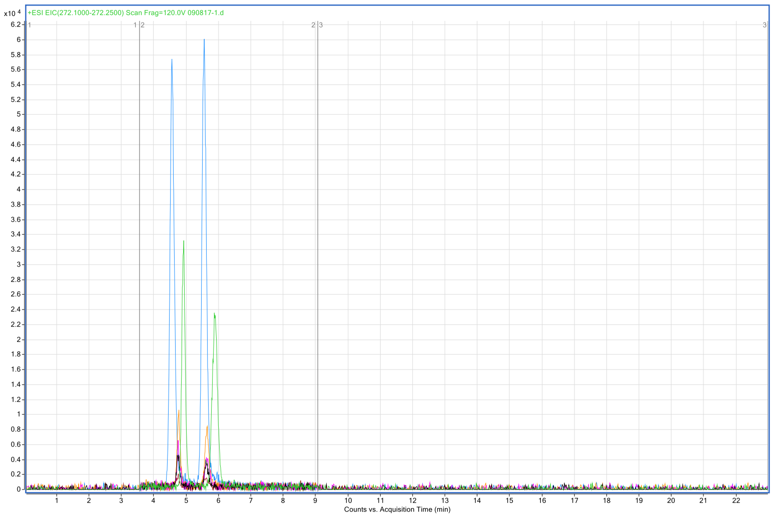

B)

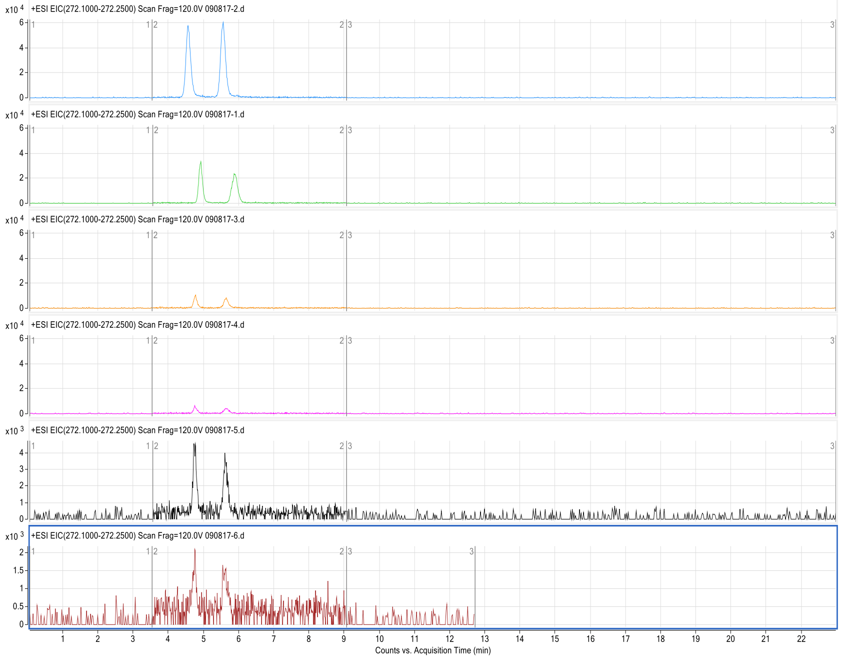

C)

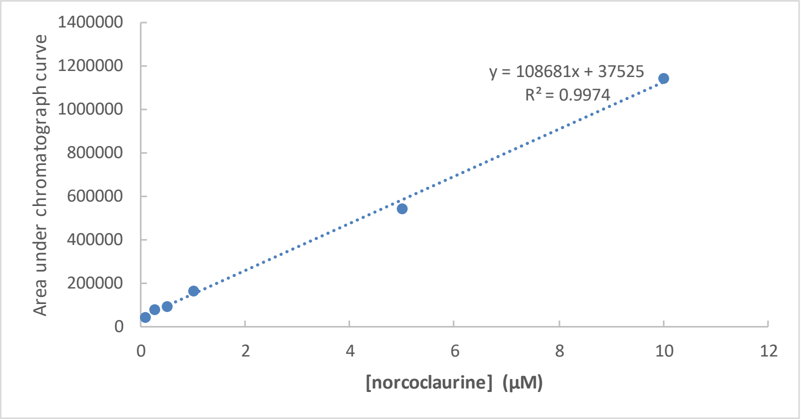

Supplementary Figure 18. Standards for norcoclaurine measurement. A) Overlay of chiral QTOF-LCMS chromatograms showing peaks for racemic norcoclaurine standards. B) Separated chromatograms corresponding to the overlay, in order from latest to earliest time point. C) Standard curve of norcoclaurine for determining absolute concentrations.

Name	Construct	Description
CEN.PK2-1D	n/a	*S. cerevisiae* strain obtained from Euroscarf
Enc	pENC1	pENC1 transformed into CEN.PK2-1D
Enc-mN-TP	pENC2	pENC2 transformed into CEN.PK2-1D, used only for demonstrating co-purification by SDS-PAGE
Enc-mNPEST-TP	pENC3	pENC3 transformed into CEN.PK2-1D, used only for demonstrating co-purification by SDS-PAGE
Enc-Venus	pENC4	pENC4 transformed into CEN.PK2-1D, used only for demonstrating co-purification by SDS-PAGE
Enc+mNPEST-TP	pENC1 and TDH3p-mNeon-PEST-TP-SYNt-KanMX4	Enc with integrated mNPEST-TP for protein stabilization studies
Enc+mNPEST	pENC1 and TDH3p-mNeon-PEST-SYNt-KanMX4	Enc with integrated mNPEST as negative control for protein stabilization studies
Enc+Ven-TP	pENC1 and TDH3p-VenN-TP-ENO2t-TEF1p-VenC-TP-SYNt-KanMX4	Enc with integrated split-Venus for protein co-localization studies
Enc+Ven-NoTPN	pENC1 and TDH3p-VenN-ENO2t-TEF1p-VenC-TP-SYNt-KanMX4	Enc with integrated split-Venus with no TP for Ven-N as negative control for protein co-localization studies
Enc+Ven-NoTPC	pENC1 and TDH3p-VenN-TP-ENO2t-TEF1p-VenC-SYNt-KanMX4	Enc with integrated split-Venus with no TP for Ven-C as negative control for protein co-localization studies
Empty+Ven-TP	pAG423GAL-ccdB and TDH3p-VenN-TP-ENO2t-TEF1p-VenC-TP-SYNt-KanMX4	Enc with integrated split-Venus with no EncA gene as negative control for protein co-localization studies
Enc+ARO10-TP	pENC1 and TEF1p-ARO10-TP-SYNt-KanMX4	Enc with integrated ARO10-TP for enzymatic catalysis studies
Enc+ARO10	pENC1 and TEF1p-ARO10-SYNt-KanMX4	Enc with integrated ARO10 as negative control for enzymatic catalysis studies

Supplementary Table 1. List of strains used in this study. Sequences of plasmids and genomic inserts are included in the accompanying SI file.

Supplementary References

1. McHugh, C. A. *et al.* A virus capsid-like nanocompartment that stores iron and protects bacteria from oxidative stress. *EMBO J.* **33**, 1896–1911 (2014).
2. Guo, Z. & Sherman, F. Signals sufficient for 3’-end formation of yeast mRNA. *Mol. Cell. Biol.* **16**, 2772–2776 (1996).
3. Kodama, Y. & Hu, C.-D. An improved bimolecular fluorescence complementation assay with a high signal-to-noise ratio. *Biotechniques* **49**, 793–805 (2010).
