# Supplementary material for: Prokaryotic nanocompartments form synthetic organelles in a eukaryote
Source: Nat Commun. 2018 Apr 3;9:1311. doi: 10.1038/s41467-018-03768-x (PMC5882880; doi:10.1038/s41467-018-03768-x)
Supplement: Supplementary file 3 — Description of Additional Supplementary Files [file 41467_2018_3768_MOESM3_ESM.pdf]

Description of Additional Supplementary Files

File Name: Supplementary Data 1
Description: GenBank files
